# Limited evidence for blood eQTLs in human sexual dimorphism

**DOI:** 10.1186/s13073-022-01088-w

**Published:** 2022-08-11

**Authors:** Eleonora Porcu, Annique Claringbould, Antoine Weihs, Kaido Lepik, BIOS Consortium, Tom G. Richardson, Uwe Völker, Federico A. Santoni, Alexander Teumer, Lude Franke, Alexandre Reymond, Zoltán Kutalik

**Affiliations:** 1 Center for Integrative Genomics, University of Lausanne, Lausanne, Switzerland; 2 Swiss Institute of Bioinformatics, Lausanne, Switzerland; 3 University Center for Primary Care and Public Health, Lausanne, Switzerland; 4 University Medical Centre Groningen, Groningen, the Netherlands; 5 Structural and Computational Biology Unit, European Molecular Biology Laboratories (EMBL), Heidelberg, Germany; 6 Department of Psychiatry and Psychotherapy, University Medicine Greifswald, Greifswald, Germany; 7 Institute of Computer Science, University of Tartu, Tartu, Estonia; 8 Estonian Genome Centre, Institute of Genomics, University of Tartu, Tartu, Estonia; 9 MRC Integrative Epidemiology Unit (IEU), Population Health Sciences, Bristol Medical School, University of Bristol, Bristol, UK; 10 Novo Nordisk Research Centre Oxford, Old Road Campus, Oxford, OX3 7DQ UK; 11 Interfaculty Institute for Genetics and Functional Genomics, University Medicine Greifswald, Greifswald, Germany; 12 DZHK (German Centre for Cardiovascular Research), partner site Greifswald, Greifswald, Germany; 13 Endocrine, Diabetes, and Metabolism Service, Centre Hospitalier Universitaire Vaudois (CHUV), Lausanne, Switzerland; 14 Faculty of Biology and Medicine, University of Lausanne, Lausanne, Switzerland; 15 Institute for Community Medicine, University Medicine Greifswald, Greifswald, Germany; 16 Department of Computational Biology, University of Lausanne, Lausanne, Switzerland

## Abstract

**Background:**

The genetic underpinning of sexual dimorphism is very poorly understood. The prevalence of many diseases differs between men and women, which could be in part caused by sex-specific genetic effects. Nevertheless, only a few published genome-wide association studies (GWAS) were performed separately in each sex. The reported enrichment of expression quantitative trait loci (eQTLs) among GWAS-associated SNPs suggests a potential role of sex-specific eQTLs in the sex-specific genetic mechanism underlying complex traits.

**Methods:**

To explore this scenario, we combined sex-specific whole blood RNA-seq eQTL data from 3447 European individuals included in BIOS Consortium and GWAS data from UK Biobank. Next, to test the presence of sex-biased causal effect of gene expression on complex traits, we performed sex-specific transcriptome-wide Mendelian randomization (TWMR) analyses on the two most sexually dimorphic traits, waist-to-hip ratio (WHR) and testosterone levels. Finally, we performed power analysis to calculate the GWAS sample size needed to observe sex-specific trait associations driven by sex-biased eQTLs.

**Results:**

Among 9 million SNP-gene pairs showing sex-combined associations, we found 18 genes with significant sex-biased *cis*-eQTLs (FDR 5%). Our phenome-wide association study of the 18 top sex-biased eQTLs on >700 traits unraveled that these eQTLs do not systematically translate into detectable sex-biased trait-associations. In addition, we observed that sex-specific causal effects of gene expression on complex traits are not driven by sex-specific eQTLs. Power analyses using real eQTL- and causal-effect sizes showed that millions of samples would be necessary to observe sex-biased trait associations that are fully driven by sex-biased *cis*-eQTLs. Compensatory effects may further hamper their detection.

**Conclusions:**

Our results suggest that sex-specific eQTLs in whole blood do not translate to detectable sex-specific trait associations of complex diseases, and vice versa that the observed sex-specific trait associations cannot be explained by sex-specific eQTLs.

**Supplementary Information:**

The online version contains supplementary material available at 10.1186/s13073-022-01088-w.

## Background

Men and women exhibit sexual dimorphism. Clear examples of sex-biased traits are anthropometric features. However, biological differences between sexes are not limited to physical traits: sex differences are also evident in incidence, prevalence, and severity across diseases. For example, women are much more likely to develop autoimmune [1], while men are more likely to develop cardiovascular diseases [2].

Despite the widespread nature of these sexual differences and their noteworthy implications for medical research and treatments, little is known about their underlying biology in complex traits. While the sex chromosomes play key roles in sexual dimorphism, genome-wide association studies (GWAS) have identified dozens of autosomal genetic variants showing sex-specific effects [3–10], suggesting that part of the phenotypic differences might be due to accumulation of genetic variants present in both sexes at the same frequency [11], but acting in a different manner in males and females.

The strong enrichment of expression quantitative trait loci (eQTLs) among complex trait-associated loci [12–15] suggests that gene expression might be an appealing intermediate phenotype for the understanding of the biological mechanism behind SNP-trait associations. Towards this goal, several transcriptome-wide association studies (TWASs) integrating GWAS and eQTLs were proposed to identify genes whose expression is significantly associated to complex traits [16–18]. As these studies pointed to many genetic loci where variants exert their effect on phenotypes through gene expression, it is reasonable to think that sex-specific associations found by GWAS could be driven by sexual dimorphism in gene expression regulation, meaning that sex differences in eQTL effects might underlie the sex-specific GWAS associations.

Previous studies that explored this hypothesis revealed that sex-biased eQTLs are associated with traits known to exhibit sex differences, including body mass index, blood pressure, lipid traits, breast cancer, and several autoimmune diseases [19–21]. However, studies characterizing sex-biased eQTLs have reported only few significant associations with the majority failing to replicate across studies. The lack of sex-biased eQTLs may suggest that the genetic control of gene expression does not substantially differ across the sexes [22] or can be due to the low power of the previous studies [23].

Here we performed a genome-wide analysis of sex-specific whole blood RNA-seq eQTLs from 3447 European individuals included in BIOS Consortium, and sought to replicate in an independent European cohort. To assess the potential contribution of sex-biased eQTLs to sex differences in complex traits, we performed PheWAS in >700 phenotypes from UKBiobank (UKB) [24] and investigate if sex-biased eQTLs translate to sex-biased trait-associations. Furthermore, we performed sex-specific transcriptome-wide Mendelian randomization (TWMR) analyses [18] combining sex-specific eQTL and GWAS data to detect sex-biased causal effect of gene expression on sexual dimorphic traits. Finally, we performed power analysis to calculate the GWAS sample size needed to observe sex-specific trait associations driven by sex-biased eQTLs.

## Methods

### Study sample

The Biobank-based Integrative Omics Study (BIOS, http://www.bbmri.nl/acquisition-use-analyze/bios/) Consortium has been set up in an effort of several Dutch biobanks to create a homogenized dataset with different levels of “omics” data layers. Genotyping was performed in each cohort separately, as described before: LifeLines DEEP [25], Leiden Longevity Study [26, 27], Netherlands Twin Registry [28], Rotterdam Study [29, 30], and Prospective ALS Study Netherlands [31]. All genotypes were imputed to the Haplotype Reference Consortium [32] using the Michigan imputation server [33].

Here, we briefly describe each cohort.

*CODAM (N=183)*: The Cohort on Diabetes and Atherosclerosis Maastricht (CODAM) is a group of individuals with a slightly increased risk of cardiometabolic disease selected from a population-based cohort [34]. Individuals in CODAM are of European descent and older than 40 years of age. They have either an increased BMI (>25), a family history of type 2 diabetes, previous gestational diabetes and/or glycosuria, or they use medication to treat hypertension.

*LLD (N=1,203)*: LifeLines DEEP (LLD) is a population-based longitudinal cohort study that includes questionnaire-based and clinical data of 167,729 individuals living in the three Northernmost provinces of the Netherlands. The study specifically focuses on families and employs a three-generational design. LLD is a subset of 1500 unrelated LifeLines participants who consented to further investigation of their genetics, gene expression, methylation, gut microbiome, and exhaled breath metabolomics.

*LLS (N=650)*: The Leiden Longevity Study (LLS) cohort studies families with individuals that reach a high age without health problems. At least two long-lived siblings (men > 88 years, women > 90 years) were required to be alive at the time of ascertainment, and their children and grandchildren are also included in the study. A total of 944 siblings from 421 European-descent families were recruited with 1671 of their offspring and 744 partners.

*NTR (N=482)*: The Netherlands Twin Register was set up in 1987 (https://tweelingenregister.vu.nl) to recruit Dutch mono- and dizygotic twins and their families. The NTR investigates health and lifestyle [35]. Twins and their relatives complete questionnaires and provide clinical measurements. From 2004 onwards, a subset of participants were asked to donate blood in order to create a biobank. Blood samples were used for genotyping, DNA and RNA isolation and to biomarker studies [36, 37]. We selected one individual from each twin pair for our study.

*RS (N=751)*: The Rotterdam Study [30] is a single-center, prospective population-based cohort study conducted in Rotterdam, the Netherlands. Subjects were included in different phases from the start of the study in 1998, with a total of 14,926 men and women aged 45 years and over included as of late 2008. The main objective of the Rotterdam Study is to investigate the prevalence and incidence of risk factors for chronic diseases to contribute to better prevention and treatment of such diseases in the elderly.

*PAN (N=173)*: PAN is a prospective study for patients suffering from amyotrophic lateral sclerosis (ALS). Since 2006, PAN aims to include all Dutch patients with ALS and similar phenotypes to correlate potential lifestyle, genetic, and environmental risk factors with the onset and prognosis of ALS (https://www.als-centrum.nl/kennisplatform/prospectieve-als-studie-nederland-pan/). To date, 3400 patients have been included, and genotypes and expression data have been generated for a subset of these patients.

RNA-seq gene expression data was generated in The Human Genotyping facility (HugeF, Erasmus MC, Rotterdam, the Netherlands, http://www.blimdna.org). RNA-seq extraction and processing has been described before for a subset of the data [38]. Briefly, RNA was extracted from whole blood and paired-end sequenced using Illumina HiSeq 2000. Reads were aligned using STAR 2.3.0e [39] while masking common (MAF > 0.01) SNPs from the Genome of the Netherlands [40]. Gene-level expression was quantified using HTseq [41]. FastQC (http://www.bioinformatics.babraham.ac.uk/projects/fastqc/) was used to check quality metrics, and we removed individuals with < 70% of reads mapping to exons (exon mapped/genome).

We followed the sample inclusion, quality control, and covariate removal previously described in eQTLGen [42] to facilitate direct comparison of the general eQTLs with the sex-biased effects. We included only unrelated individuals in this analysis and removed population outliers by filtering out samples with >3 standard deviations from the average heterogeneity score. The gene expression data from all cohorts combined was corrected for a subset of the first 25 principal components (PCs) that were not associated with genetics (see Additional file 1: Fig. S1 for selection), to control for unmeasured variation while avoiding the removal of eQTL effects.

We stratified the samples by sex and performed the *cis*-eQTL mapping using a pipeline described previously [43]. In brief, the pipeline takes a window of 1Mb upstream and 1Mb downstream around each SNP to select genes or expression probes to test, based on the center position of the gene or probe. The association between these SNP-gene combinations was calculated using a Spearman correlation in each sex separately.

### Differential gene expression and variance analysis

Differential expression analysis was performed without considering genotypes, to identify genes with sex-specific expression (FDR 5%). For each gene, to test the difference in mean in the two sexes, we used the *t* statistics

$$t=\frac{\mu_F-{\mu}_M}{\sqrt{\sigma_F^2+{\sigma}_M^2}}$$

where *μ* and *σ* are the mean and the standard error, respectively.

To detect genes with sex-specific expression variance, we tested for difference in variance between females (F) and males (M) using an *F*-test, i.e.,

$$F=\frac{\sigma_F^2}{\sigma_M^2}\sim F\left({N}_F,{N}_M\right)$$

### Sex-specific eQTL effects

To identify SNP-gene pairs with sex difference, we computed *P*-values (*P*_diff_) testing for difference between the standardized men-specific and women-specific *β*_eQTL_-estimates, with corresponding standard errors and using the t statistic

$$t=\frac{\beta_{\mathrm{eQTL}\left(\mathrm{F}\right)}-{\beta}_{\mathrm{eQTL}\left(\mathrm{M}\right)}}{\sqrt{\frac{1}{N_{\mathrm{eQTL}\left(\mathrm{F}\right)}}+\frac{1}{N_{\mathrm{eQTL}\left(\mathrm{M}\right)}}}}$$

We selected 462 sex-specific SNP-gene pairs at an FDR of 5% across all the pairs tested.

### Impact of PC correction on sex-specific eQTL detection

We tested if the PCs that we removed from the gene expression matrix were associated with sex by calculating if there was a mean difference between the sexes for each of the PCs using the Wilcoxon test with correction for 17 PCs. As some PCs were associated with sex, we next investigated whether the sex-specific gene expression distributions were affected by PC removal. We studied the gene expression distribution for the 18 significant eQTL genes and tested if the variation between men and women had changed before and after PC removal using Levene’s test.

### Sex-specific cell type distribution

eQTLs might arise from differences in cell type composition, rather than intracellular gene expression changes resulting from a genetic variant. The 33 cell counts were imputed using RNA expression and a reference panel of blood cell counts as implemented in the Decon2 package and previously described [44]. We used a Wilcoxon test to evaluate if cell type proportions were different for men and women (*P*_Wilcoxon_ < 0.05/33). Additional file 1: Fig. S1 shows that much of the cell count variation was captured by the PCs that were regressed out of the expression data.

### Replication analyses in SHIP-Trend

#### Study population

The Study of Health in Pomerania (SHIP-Trend) is a population-based cohort study in West Pomerania, a region in the northeast of Germany, assessing the prevalence and incidence of common population-relevant diseases and their risk factors. Baseline examinations for SHIP-Trend were carried out between 2008 and 2012, comprising 4420 participants aged 20 to 81 years. Study design and sampling methods were previously described [45].

#### Genotyping

Nonfasting blood samples were drawn from the cubital vein in the supine position. The samples were taken between 07:00 AM and 04:00 PM, and serum aliquots were prepared for immediate analysis and for storage at −80 °C in the Integrated Research Biobank (Liconic, Liechtenstein). A subset of the SHIP-Trend samples was genotyped using the Illumina Human Omni 2.5 array. Hybridization of genomic DNA was done in accordance with the manufacturer’s standard recommendations at the Helmholtz Zentrum München. Genotypes were determined using the GenomeStudio Genotyping Module v1.0 (GenCall algorithm, https://support.illumina.com/array/array_software/genomestudio/documentation.html). Arrays with a genotyping call rate <94%, duplicates (based on estimated IBD), and mismatches between reported and genotyped sex were removed, leaving 986 arrays for subsequent analyses. Imputation of genotypes was performed using the HRCv1.1 reference panel [32] and the Eagle [46] and minimac3 [33] software implemented in the Michigan Imputation Server for pre-phasing and imputation [33], respectively. SNPs with a Hardy-Weinberg-Equilibrium *P*-value <0.0001, a call rate <0.95, or monomorphic SNPs were removed before imputation, as well as SNPs having position mapping problem from genome build b36 to b37, duplicate IDs, or with inconsistent reference site alleles.

#### Whole-blood transcriptome analysis

RNA was prepared from whole blood under fasting conditions in PAXgene tubes (BD) using the PAXgene Blood miRNA Kit (Qiagen, Hilden, Germany). For SHIP-Trend, this was done on a QIAcube according to protocols provided by the manufacturer (Qiagen). To ensure a constant high quality of the RNA preparations, all RNA samples were analyzed using RNA 6000 Nano LabChips (Agilent Technologies) on a 2100 Bioanalyzer (Agilent Technologies) according to the manufacturer’s instructions. Using the Illumina TotalPrep-96 RNA Amp Kit (Ambion), 500ng of RNA was reverse transcribed into cRNA, and biotin-UTP-labeled. Three thousand nanograms of cRNA was hybridized to the Illumina HumanHT-12 v3 Expression BeadChips, followed by washing steps as described in the Illumina protocol. Processing of the SHIP-Trend DNA and RNA samples was performed at the Helmholtz Zentrum München. For gene expression analysis, raw intensity data generated with the expression arrays were exported from Illumina’s GenomeStudio V 2010.1 Gene Expression Module to the R environment and processed (quantile normalization and log2-transformation) with the lumi 1.12.4 package from the Bioconductor open source software (http://www.bioconductor.org/). Details on quality control and data preparation are described in Schurmann et al. [47].

#### Analyses

In SHIP-Trend, the sex-stratified eQTL analysis was performed on 991 subjects (555 females). Linear regression analysis of eQTL was carried out in R. To adjust for confounding effects, all models were adjusted for the first 50 principal components, calculated based on the gene-expression data, with none being highly correlated with any of the SNPs (all spearman-correlation coefficient *P*-values > 1e−12).

### Sex-specific GWAS effects

To identify SNPs with sex-difference in the 39 phenotypes we found associated with the lead sex-biased eQTLs, we downloaded the summary statistics of the sex-stratified GWAS available at http://www.nealelab.is/uk-biobank/.

We computed *P*-values (*P*_diff_) testing for a difference between the men-specific and women-specific *β*_*GWAS*_-estimates, with corresponding standard errors and using the *t* statistic

$$t=\frac{\beta_{\mathrm{GWAS}\left(\mathrm{F}\right)}-{\beta}_{\mathrm{GWAS}\left(\mathrm{M}\right)}}{\sqrt{SE_{(F)}^2+{SE}_{(M)}^2}}$$

All the statistical analysis were performed using R version 3.6.0 software (The R Foundation).

### Phenome-wide association analysis (PheWAS) in UKB

We queried GeneATLAS (http://geneatlas.roslin.ed.au.uk/) for trait associations with the 18 lead sex-biased eQTLs. Summary statistics for traits associated with each queried variant were downloaded from GeneATLAS.

### Transcriptome-wide Mendelian randomization (TWMR) analyses

Univariable transcriptome-wide Mendelian randomization (TWMR) [18] analyses were conducted to estimate the causal effect of gene expression on waist-to-hip ratio (WHR), testosterone, and educational attainment. First, we ran TWMR separately in the two sexes, combining sex-specific eQTL data from BIOS Consortium and sex-specific GWAS summary statistics from UKBiobank (http://www.nealelab.is/uk-biobank/). We tested 4363 and 3692 genes with at least 3 independent significant (*P*<0.001) eQTLs in females and males, respectively. Second, for testosterone, we ran again TWMR separately in the two sexes but using sex-specific GWAS summary statistics and sex-combined eQTL data from eQTLGen Consortium. We tested 7982 genes with at least 3 independent significant (*P*<1.83E−05) eQTLs in both sexes.

### Power analyses

We performed power analyses to calculate the probability that sex-specific SNPs found by GWAS are driven by sex-specific eQTLs. Using real observed data, we tested the power to detect a significant difference in *β*_GWAS_ in males and females starting from the difference observed in *β*_eQTL_ and the causal effect of the gene expression on the phenotype calculated by TWMR (α_TWMR_).

If the association of a SNP in a given phenotype is driven by an eQTL, then we have

$${\beta}_{\mathrm{GWAS}\left(\mathrm{Females}\right)}={\alpha}_{\mathrm{TWMR}\left(\mathrm{Females}\right)}\times {\beta}_{\mathrm{eQTL}\left(\mathrm{Females}\right)}$$

$${\beta}_{\mathrm{GWAS}\left(\mathrm{Males}\right)}={\alpha}_{\mathrm{TWMR}\left(\mathrm{Males}\right)}\times {\beta}_{\mathrm{eQTL}\left(\mathrm{Males}\right)}$$

Let’s suppose that the effect of the gene expression on the phenotype is the same in the two sexes

$${\alpha}_{\mathrm{TWMR}\left(\mathrm{F}\right)}={\alpha}_{\mathrm{TWMR}\left(\mathrm{M}\right)}={\alpha}_{\mathrm{TWMR}}$$

Then

$${\beta}_{\mathrm{GWAS}\left(\mathrm{F}\right)}-{\beta}_{\mathrm{GWAS}\left(\mathrm{M}\right)}={\alpha}_{\mathrm{TWMR}}\times \left({\beta}_{\mathrm{eQTL}\left(\mathrm{F}\right)}-{\beta}_{\mathrm{eQTL}\left(\mathrm{M}\right)}\right).$$

Since ${\hat{\beta}}_{\mathrm{GWAS}\left(\mathrm{F}\right)}\sim N\left({\beta}_{\mathrm{GWAS}\left(\mathrm{F}\right)},\frac{1}{N_{\mathrm{GWAS}\left(\mathrm{F}\right)}}\right)$ and ${\hat{\beta}}_{\mathrm{GWAS}\left(\mathrm{M}\right)}\sim N\left({\beta}_{\mathrm{GWAS}\left(\mathrm{M}\right)},\frac{1}{N_{\mathrm{GWAS}\left(\mathrm{M}\right)}}\right)$, our statistics *t* follows a normal distribution when *N* is large,

$$t=\frac{{\hat{\beta}}_{\mathrm{GWAS}\left(\mathrm{F}\right)}-{\hat{\beta}}_{\mathrm{GWAS}\left(\mathrm{M}\right)}}{\sqrt{\frac{1}{N_{\mathrm{GWAS}\left(\mathrm{F}\right)}}+\frac{1}{N_{\mathrm{GWAS}\left(\mathrm{M}\right)}}}}\sim N\left(\frac{\beta_{\mathrm{GWAS}\left(\mathrm{F}\right)}-{\beta}_{\mathrm{GWAS}\left(\mathrm{M}\right)}}{\sqrt{\frac{1}{N_{\mathrm{GWAS}\left(\mathrm{F}\right)}}+\frac{1}{N_{\mathrm{GWAS}\left(\mathrm{M}\right)}}}},1\right)$$

We tested the hypothesis *H*_0_ : *ϑ* = *β*_GWAS(F)_ − *β*_GWAS(M)_ = 0 against *H*_1_ : *ϑ* = *β*_GWAS(F)_ − *β*_GWAS(M)_ ≠ 0.

Using the genome-wide significance threshold, *H*_0_ will be rejected if

$$\mid t\mid >{\Phi}^{-1}\left(2.5\times {10}^{-8}\right).$$

Then, the power to detect *β*_GWAS(F)_ − *β*_GWAS(M)_ = *ϑ* is

$$P\left(\left|\frac{\alpha_{\mathrm{TWMR}}\times \left({\beta}_{\mathrm{eQTL}\left(\mathrm{F}\right)}-{\beta}_{\mathrm{eQTL}\left(\mathrm{M}\right)}\right)}{\sqrt{\frac{1}{N_{\mathrm{GWAS}\left(\mathrm{F}\right)}}+\frac{1}{N_{\mathrm{GWAS}\left(\mathrm{M}\right)}}}}\right|>5.43\right)=2\times \left(1-\Phi\ \left(5.43-\frac{\alpha_{\mathrm{TWMR}}\times \left({\beta}_{\mathrm{eQTL}\left(\mathrm{F}\right)}-{\beta}_{\mathrm{eQTL}\left(\mathrm{M}\right)}\right)}{\sqrt{\frac{1}{N_{\mathrm{GWAS}\left(\mathrm{F}\right)}}+\frac{1}{N_{\mathrm{GWAS}\left(\mathrm{M}\right)}}}}\right)\right)$$

We performed the power analyses using the difference of *β*_eQTL_ effects observed in the 18 top sex-specific eQTLs and in 6 quantiles extracted from the distribution of significant causal effects estimated by TWMR for WHR.

Using the same statistics, we calculated the sample size to observe *β*_GWAS(F)_ − *β*_GWAS(M)_ = *ϑ* with power >0.8.

All the analysis were performed using R version 3.6.0 software (The R Foundation).

## Results

### Sex-biased eQTL analyses

First, we performed a genome-wide analysis of whole blood RNA-seq eQTLs to identify autosomal sex-biased eQTLs, i.e., SNPs whose effect on expression differs in magnitude between men and women. We analyzed eQTLs separately for 1519 men and 1928 women collected by the BIOS Consortium (http://www.bbmri.nl/acquisition-use-analyze/bios/). To reduce the number of tests, we restricted our analyses to autosomal variants previously detected as *cis*-eQTLs (FDR 5%) by the sex-combined analysis of the eQTLGen Consortium [42] and included in the UK10K reference panel [48].

To test the reliability of the BIOS data, we combined the results from the two sexes in a meta-analysis and compared them with those obtained by the eQTLGen Consortium (*N*=31,684, sex-combined results). We observed a high correlation between the betas obtained in the two studies (*r*^*2*^=0.9).

In total, we tested 8,739,806 SNP-gene pairs (involving 3,142,796 SNPs and 16,874 genes) for sex-interaction. Among those, we replicated 6,264,342 and 6,708,475 SNP-gene pairs associations as significant (FDR 5%) in men and women respectively (${\pi}_{1_{men}}=0.12,{\pi}_{1_{women}}=0.10$). We found 16,069 eGenes (genes with at least one *cis*-eQTL at FDR 5%) shared between the two sexes, while 215 and 353 eGenes were detected only in men or women, respectively.

### Identification of sex-specific *cis*-eQTLs

To identify sex-biased eQTLs, we tested the difference in the effects calculated for the two sexes separately (see the “Methods” section).

**Fig. 1:**
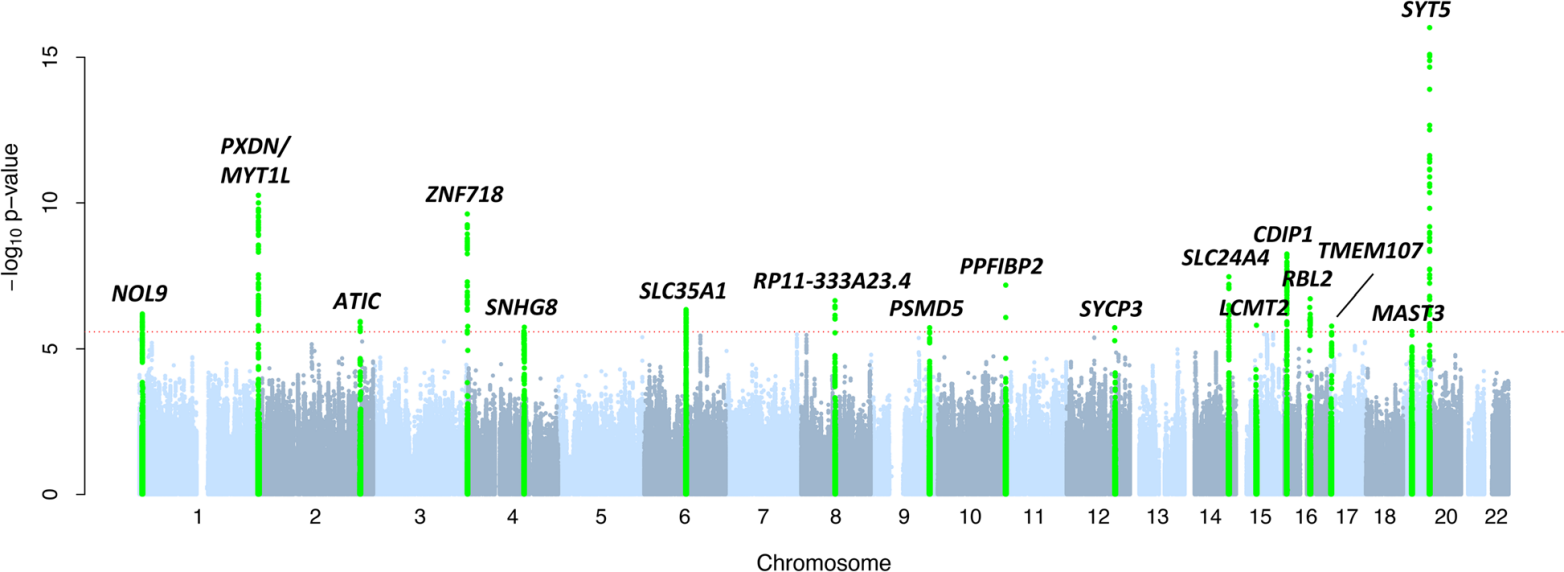
Manhattan plot of sex-specific eQTLs. The figure summarizes the results of our sex-specific eQTL discovery scan. SNP-gene pairs are plotted on the *x*-axis according to the SNP position on each autosomal chromosome in alternating light and dark blue against the *P*-values obtained upon testing for sex difference between effects in men and women (shown as –log10(Pdiff)). The red dotted line marks the 5% FDR threshold significance level (*P*_diff_=2.6×10^−6^), and SNPs in loci exceeding this threshold are highlighted in green

We identified 462 SNP-gene associations showing significantly different effects in men and women (FDR 5%, *P*_diff_<2.6×10^−06^). These sex-biased eQTLs cluster in 18 sex-biased eGenes (Fig. 1 and Additional file 2: Table S1). By analyzing only *cis*-eQTLs with a significant sex-combined effect (in the eQTLGen Consortium), we favored eQTLs that show the same direction but different magnitude of effect in men and women, as we posit that significant eQTLs showing opposite direction of effects in the two sexes would not have been detected in sex-combined analyses. Consistent with this hypothesis, all 462 sex-biased eQTLs show the same direction but different magnitude of effects in both sexes.

Among the 18 sex-biased eGenes, 5 and 12 are men- and women-biased, respectively. For one eGene, *ZNF718*, some variants are biased for males and others for females (Additional file 1: Fig. S2). Looking at the linkage disequilibrium (LD) between these eQTLs, we observed that the variants belong to two different LD blocks (Additional file 1: Fig. S3).

To test if we could have a better enrichment if we looked at the hormone-to-TF binding targets, we extracted the positions of the binding sites of six estrogen receptor genes reported in a previous study [49]: *ESR1*, *PGR*, *GREB1*, *MYC*, *GATA3*, and *CTSD*. Out of the seven eQTLs (two for *ESR1* and five for *CTSD*) residing in the binding sites, none of those shows any difference in the two sexes (*P*_diff_>0.05) (Additional file 2: Table S2).

To determine if the difference of eQTL effects observed between men and women is driven by sex differences in gene expression distribution, we then compared the expression means and variances between sexes for the 18 sex-biased eGenes. While no eGene showed a significant difference in mean, we found 8 eGenes with sex-specific variance (based on FDR 5%, *P*_diff_ < 6.4 × 10^−03^ - OR=3.53, hypergeometric *P*=7.1×10^−04^) (Additional file 2: Table S3, Additional file 1: Fig. S4).

Whole blood is a mixture of heterogeneous cell types and such cell type composition differs between sexes [50, 51]. Using the Wilcoxon test, we checked for mean sex differences in cellular composition on the basis of estimated abundance of 33 cell types. We discovered significant (*P* < 0.05/33) differences for nearly all (31/33) cell types (Additional file 2: Table S4 and Additional file 1: Fig. S5). Although the abundance of cell types correlates with sex, the eGenes did not recapitulate that pattern, suggesting that the sex-biased eQTLs were not driven by the difference in cell type abundance.

To account for universal confounders of gene expression, we corrected for up to 25 principal components (PCs) of the gene expression. Although some of them were associated with sex (Wilcoxon’s test, Additional file 2: Table S5), correction for PCs did not majorly influence the gene expression distribution of the 18 sex-specific eGenes (Levene’s test of variation changed from significant (*P*_Levene_ < 0.05/18) to non-significant for only 3 genes upon PC correction (Additional file 2: Table S6 and Additional file 1: Fig. S6).

Although we did not replicate any sex-biased eQTLs reported in previous studies [19, 20] (Additional file 2: Table S7 and Additional file 2: Table S8), we sought to replicate the sex-biased eQTLs identified here in an independent European cohort, SHIP-Trend (555 females and 436 males). Out of the 18 sex-biased eGenes, 15 were measured in SHIP-Trend. We restricted our replication to the lead eQTLs showing nominal significant *P*-values (*P*<0.05) in the sex-combined analysis of SHIP-Trend to filter out potentially problematic genes (or eQTLs with incompatibly small marginal effect). In total, we tested 6 eQTLs and for 3 of them we observed a directionally consistent, nominally significant difference in effect in the two sexes (Additional file 2: Table S9).

### Sex-specific *cis*-eQTLs do not translate into sex-specific trait associations

We then performed a phenome-wide association study (PheWAS) to test if eQTL SNPs with sex-biased effect on expression levels have an effect on human phenotypes and if so, whether sex biases in gene expression regulation translate to sex-biased effects on complex traits. For each sex-biased eGene, we selected one representative eQTL with the strongest difference in effect between men and women and ran PheWAS analyses on more than 700 phenotypes from UKB.

We found that 7 of the 18 lead eQTLs were associated with 39 traits at genome-wide significant level (Additional file 2: Table S10). Interestingly all associated traits belong to two categories: either morphological (e.g., height, weight and trunk fat mass) or hematological traits (e.g., platelets, eosinophils, and whole blood cells).

**Fig. 2:**
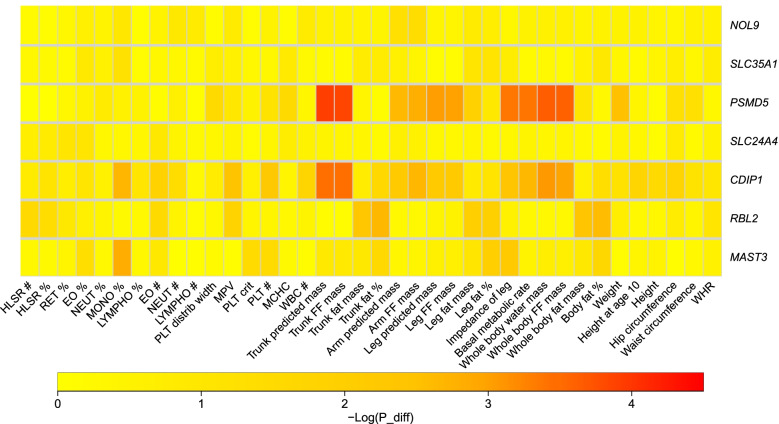
Heatmap of sex-specific trait-associated SNPs. The figure summarizes the results of sex-specific trait-associations driven by sex-specific eQTLs. For each sex-specific eGenes and for each phenotype, we plotted the minimum *P*_diff_ obtained testing for sex-difference between GWAS-effects in men and women (shown as –log_10_(*P*_diff_))

We then asked if the other 290 non-lead sex-biased eQTLs of the 7 eGenes found by PheWAS showed also a sex-biased effect on the 39 pre-selected traits (we set a Bonferroni threshold of *P*_diff_<0.05/(20×7), where 20 is the effective number of independent phenotypes among the 39). For each trait, we used the sex-specific UKB GWAS summary statistics and observed no enrichment for the sex-biased eQTLs among the sex-biased GWAS signals and found only one eGene, *PSMD5*, for which sex-biased eQTLs are likely to translate to sex-biased associations with several obesity traits (Fig. 2).

### Sex-specific complex trait associations are not driven by sex-specific eQTLs

Next, we tested whether sex-biased SNP-trait associations are driven by sex-biased gene expression regulation. For this we looked at the two most sexually dimorphic traits, waist-to-hip ratio (WHR) and testosterone levels. We identified 803 and 266 independent SNPs showing a *P*-value < 1×10^−05^ in the sex-combined GWAS for WHR and testosterone, respectively. Among those, 121 (32 for WHR and 89 for testosterone) have a significant sex difference in the effect on the two sexes (*P*_diff_ <0.05/(803+266)), but none of the 58 SNPs included in the BIOS Consortium dataset show any sex-biased eQTL effect on genes in *cis* (*P*_diff_<0.05/58) (Additional file 2: Table S11 and Additional file 2: Table S12 and Additional file 1: Fig. S7).

### Sex-biased causal effects

Next, to explore the presence of sex-biased causal effect of gene expression on complex traits, we performed sex-specific TWMR analyses combining sex-stratified eQTL and sex-stratified GWAS results. We applied such approach—as above—to testosterone levels and WHR.

For testosterone we found 33 and 10 genes significant for men and women respectively. Of note, eight and two genes were missed by the GWAS performed in the sex-stratified GWAS, i.e., in GWAS-men and GWAS-women, respectively, pointing to new associated regions (Additional file 2: Table S13). For WHR, we found 13 and 24 significant genes for men and women respectively. As see for testosterone, also for WHR our results pointed to new associated regions: seven and three genes were missed by the GWAS performed in men and women respectively (Additional file 2: Table S14).

Interestingly, we found 19 and 1 genes showing a significantly different causal effect between the two sexes in testosterone and WHR, respectively (Additional file 1: Fig. S8 and Additional file 1: Fig. S9 and Additional file 2: Table S15). While the sex-biased association with WHR was female-specific, among the 19 sex-biased genes associated with testosterone, 4 were female- and 15 male-biased, respectively.

Of note, the negative association of *SPAG1* with testosterone levels observed only in women (*P*_TWMR-women_=2.99×10^−06^) and not in men (*P*_TWMR-men_=0.18) is supported by its association with infertility in women [52]. Among the male-specific genes, we observed a positive association for *ARK1C2* (*P*_TWMR-women_=0.036 and *P*_TWMR-men_=1.43×10^−05^), already found associated with 46, XY sex reversal (OMIM #614279), which presents minimal testosterone among its symptoms.

As a negative control, we applied TWMR to educational attainment, a trait not showing sexual dimorphism and did not observe any sex-biased gene association (Additional file 1: Fig. S10).

Finally, we tested whether sex-biased causal effects are driven by sex-biased gene expression regulation and observed that none of the SNPs used as instrumental variables in TWMR showed a sex-biased effect on gene expression. In addition, we ran TWMR using sex-specific testosterone GWAS and sex-combined eQTL data and found a high correlation between the causal effects (*r*^*2*^=0.75 in females and *r*^*2*^=0.77 in males) (Additional file 1: Fig. S11), which suggests that different effects observed by TWMR are driven by sex-biased SNP-trait associations.

### Power to detect sex-specific trait-associations

Since the observed sex-biased SNP-trait associations do not seem to be driven by sex-biased eQTL effects in our data, we performed power analyses using eQTL-effect differences observed in the data of the BIOS Consortium.

If the SNP-trait association is fully mediated by gene expression, then in females (F) and males (M)

$${\beta}_{\mathrm{GWAS}\left(\mathrm{F}\right)}={\upalpha}_{\mathrm{TWMR}\left(\mathrm{F}\right)}\ast {\beta}_{\mathrm{eQTL}\left(\mathrm{F}\right)}$$

and

$${\beta}_{\mathrm{GWAS}\left(\mathrm{M}\right)}={\upalpha}_{\mathrm{TWMR}\left(\mathrm{M}\right)}\ast {\beta}_{\mathrm{eQTL}\left(\mathrm{M}\right)}$$

where *β*_GWAS_ and *β*_eQTL_ indicate the effect of the SNP on the trait and on gene expression, respectively, and α_*TWMR*_ is the causal effect of the gene expression on the trait. Assuming the same causal effect of the gene expression on the trait in both sexes (i.e., α_TWMR(M)_ = α_TWMR(F)_ = α_TWMR_), the difference of SNP effect on the trait should be

$${\beta}_{\mathrm{GWAS}\left(\mathrm{F}\right)}-{\beta}_{\mathrm{GWAS}\left(\mathrm{M}\right)}={\upalpha}_{\mathrm{TWMR}}\ast \left({\beta}_{\mathrm{eQTL}\left(\mathrm{F}\right)}-{\beta}_{\mathrm{eQTL}\left(\mathrm{M}\right)}\right)$$

**Fig. 3:**
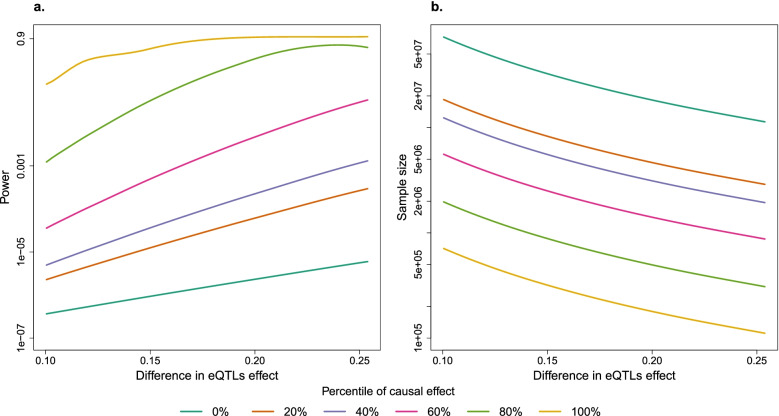
Power analysis results. **a** Plot showing the power to detect significantly different effect in sex-specific GWAS collecting 190,000 females and 170,000 males for different percentiles of causal effect. **b** Plot showing the total sample size (females + males) needed to detect significantly different effect in sex-specific GWAS at power > 80% for different percentiles of causal effect

Using the differences observed by the BIOS Consortium and the causal effects estimated for a large number of complex traits by TWMR [18], we observed that the power to detect sex-specific trait associations driven by sex-specific eQTLs is null, with an exception in the case when the largest causal effect of the gene expression on the trait observed in TWMR (100th percentile) is coupled with the largest differences in eQTL-effects observed in BIOS Consortium (Fig. 3a). We also estimated the GWAS sample size required to reach 80% power to detect differences in sex-specific GWAS. We found that, using the subset of unrelated British individuals from UKB (*N*=380K), we do not have the power to detect sex differences driven by SNPs being eQTLs for causal genes. Indeed, our results show that even when we used the 80th percentile of the distribution of the significant causal effects of the gene expression on the trait, we need one to five million individuals to detect the different effect driven by sex-biased eQTLs (Fig. 3b).

## Discussion

Notwithstanding the prominent differences in traits observed between men and women, there is little known about the role of sex-specific genetic effects. Several sex-stratified GWASs identified sex-specific genetic variants on autosomal chromosomes, which highlights that not all differences are located on the sex chromosomes [3–10]. Since many genetic variants exert their effect on complex traits through gene expression [17, 18], sex differences in eQTL effects might underlie such sex-specific GWAS associations.

In this study, we used large sex-specific eQTL data and sex-specific GWAS results to investigate the role of gene expression on the sexual dimorphism of several human phenotypes. The genome-wide analysis that aimed to identify sex-biased eQTLs confirms what was already found by previous studies, i.e., that men and women share the same common genetic control of gene expression. We think there could be different explanations to the lack of sex-specific eQTLs: (i) Sex-differential gene expression is primarily not under genomic control, but mostly influenced by hormonal differences. A similar observation was made for complex traits: while most complex traits have vastly different mean values for men and women (e.g., height), no sex-specific QTLs have been identified to date. (ii) The sample size of our study is too small to have the power to detect most sex-specific effects. Sex-differential gene expression regulation may be just as complex as it is for any complex trait. (iii) Ours and previous studies [19, 20] were focused on whole blood which is a highly heterogeneous tissue. Sex-specific eQTLs could be tissue-/cell type-specific and they might be missed in bulk data because they show sex-specific effects only in a cell type or at single cell level.

Although we identified 18 sex-biased eGenes and 7 of them were associated with traits known to exhibit sex differences, our results suggest that sex-biased eQTLs in whole blood do not translate to detectable sex-specific trait associations, and vice versa that the observed sex-biased trait associations cannot be explained by sex-biased eQTLs. While recent work revealed that ~11% of trait heritability could be explained by *cis*-eQTL regulation [53], our findings show that the sex-specific heritability is not detectably mediated by sex-specific gene expression regulation. Our extensive power analyses, performed using a range of realistic effect sizes, confirmed these observations. Indeed we demonstrated that with the current sample size used in sex-specific GWAS, we do not have the power to detect differences in sex-biased trait-associations driven by sex-biased eQTLs. Our results suggest that we will be able to explore how sex-specific gene expression regulation translates to complex traits only when GWAS will be performed on millions of individuals. It is only then that we will be able to test the existence of potential compensatory mechanisms via negative feedback loops dampening such signal propagation.

There are some limitations to this study. Firstly, although the BIOS RNA-seq dataset is relatively large (including 1918 women and 1519 men), it is limited to whole blood. Since it is known that the effect of causal genes on diseases typically act in a tissue-specific manner [54–56], the investigation of other, more relevant, tissues could be crucial to estimate larger causal effects and unravel the sex-specific associations found by the GWAS. Secondly, we did not include the X chromosome and limited our analyses to autosomal variants previously detected as significant *cis*-eQTLs by the sex-combined analysis performed by eQTLGen Consortium [42], which will be biased against discovering sex-specific effects with opposite sign in men and women. However, a previous study [57] reported consistent direction of allelic effects on gene expression across the sexes suggesting that if an eQTL is positively associated in one sex, it will have the same direction of association (or none) in the other sex. Of note, as eQTLGen Consortium data are restricted to primary *cis*-eQTLs, here we also missed all the SNPs influencing gene expression independently whose effects are masked by the effect of the corresponding top eQTL and can be only found by conditional analysis [58]. These simplifications were necessary to keep multiple testing burden to the minimum and hence maximize power in the small sex-specific eQTL study.

Moreover, an additional limitation is certainly the absence of correction for any confounding factor. Indeed, both exogeneous factors—such as environmental exposures—and endogenous factors—such as hormones and reproductive events—could influence gene expression and mask, or bias, sex-specific effects [59]. Both the complex trait and the gene expression data were measured at a single timepoint, rendering the analysis blind to time-variant (e.g., periodic) mechanisms, which may be crucial for hormone-related modifier effects.

Finally, although we used the biggest sex-specific GWAS results, we convincingly show that the currently available sample size is too small to reach the statistical power necessary for detecting sex-specific trait-association mediated by sex-specific blood eQTLs. Also, to avoid false positives, we were conservative in our analyses through extensive data quality controls and PC corrections, that possibly reduced power and masked sex-specific associations. Our study highlights the importance of interrogating other types of data: as several studies have shown that eQTL effects can be cell type-specific [60, 61], upcoming single-cell eQTL datasets [62] might be essential in identifying sex- and cell-type specific effects and unravel the biological mechanism behind sexual dimorphism. Alternatively, if sexual dimorphism of complex traits is not driven by gene expression changes, we might need to explore other types of omics data to gain a deeper insight into the molecular underpinnings of sex differences in complex diseases.

## Conclusions

In this work, we combined the largest-to-date sex-specific eQTL and GWAS data to investigate the role of gene expression in the sexual dimorphism of several human phenotypes. Our results show that the sex-specific heritability is not detectably mediated by sex-specific gene expression regulation in whole blood. This observation highlights the importance to explore other types of omics data to understand the genetics of sexually dimorphic traits.

## Supplementary Information

**Additional file 1: Figure S1.** Correlation between the principal components (PCs) and imputed and measured cell counts. **Figure S2.** Comparison between the effects in the two sexes of the eQTLs in the 18 sex-specific eGenes. **Figure S3.** LD structure of *ZNF718* region**. Figure S4.** Distribution of the expression values for the 18 sex-biased eGenes. **Figure S5.** Cell type distribution in men and women. **Figure S6.** Distribution of the expression values after and before the PC correction. **Figure S7.** QQ-plot for the 58 SNPs included in the BIOS Consortium dataset showing sex-biased effect in WHR or testosterone. **Figure S8.** Miami plot for testosterone measured in UKBiobank. **Figure S9.** Miami plot for waist-to-hip ratio (WHR) measured in UKBiobank. **Figure S10.** Miami plot for educational attainment measured in UKBiobank. **Figure S11.** Comparison of TWMR-causal effects estimated for WHR using sex-specific and combined eQTLs data.

**Additional file 2: Table S1.** Sex-specific eQTLs. **Table S2.** Summary statistics for eQTLs in binding sites for estrogen receptor genes. **Table S3.** Gene level summary statistics. **Table S4.** Wilcoxon test *P*-values for mean sex differences in cellular composition. **Table S5.** Wilcoxon test *P*-values for mean sex differences in principal components. **Table S6.** Levene test *P*-values for mean sex differences before and after correcting for principal components. **Table S7.** BIOS results for the sex-biased eQTLs found in Yao et al [PMID: 24242183]. **Table S8**. BIOS results for the sex-biased eQTLs found in Kukurba et al [PMID: 27197214]. **Table S9.** Replication analyses results - SHIP-Trend cohort. **Table S10**. PheWAS for top sex-specific eQTLs. **Table S11.** Independent SNPs associated with WHR. **Table S12.** Independent SNPs associated with testosterone. **Table S13.** Sex-stratified TWMR results for testosterone. **Table S14.** Sex-stratified TWMR results for WHR. **Table S15.** Sex specific causal genes for WHR and testosterone.

## Data Availability

The BIOS RNA-seq, DNA methylation, sex, age, and cell count data has been deposited previously in the EGA (https://www.ebi.ac.uk/ega/studies/EGAS00001001077) [63]. Per-cohort individual-level genotype and gene expression data are governed by respective biobanks and access can be requested according to procedures established by each biobank, with relevant restrictions applying as imposed by the IRB or local legislation. Researchers can fill out a data request form and sign the BIOS Code of Conduct, as described on https://www.bbmri.nl/acquisition-use-analyze/bios. The SHIP-Trend expression dataset is available at GEO (Gene Expression Omnibus) public repository under the accession GSE36382: 991 samples were available for analysis [64]. The genetic data and the sex information of the SHIP-Trend study cannot be made publicly available as informed consent for sharing this data was not provided by the study participants, but it can be accessed through a data application form available at https://fvcm.med.uni-greifswald.de/ for researchers who meet the criteria for access to confidential data.
