# Supplementary material for: Limited evidence for blood eQTLs in human sexual dimorphism
Source: Genome Med. 2022 Aug 11;14:89. doi: 10.1186/s13073-022-01088-w (PMC9373355; doi:10.1186/s13073-022-01088-w)
Supplement: Supplementary file 1 — Additional file 1: Figure S1. Correlation between the principal components (PCs) and imputed and measured cell counts. Figure S2. Comparison between the effects in the two sexes of the eQTLs in the 18 sex-specific eGenes. Figure S3. LD structure of ZNF718 region. Figure S4. Distribution of the expression values for the 18 sex-biased eGenes. Figure S5. Cell type distribution in men and women. Figure S6. Distribution of the expression values after and before the PC correction. Figure S7. QQ-plot for the 58 SNPs included in the BIOS Consortium dataset showing sex-biased effect in WHR or testosterone. Figure S8. Miami plot for testosterone measured in UKBiobank. Figure S9. Miami plot for waist-to-hip ratio (WHR) measured in UKBiobank. Figure S10. Miami plot for educational attainment measured in UKBiobank. Figure S11. Comparison of TWMR-causal effects estimated for WHR using sex-specific and combined eQTLs data. [file 13073_2022_1088_MOESM1_ESM.docx]

**
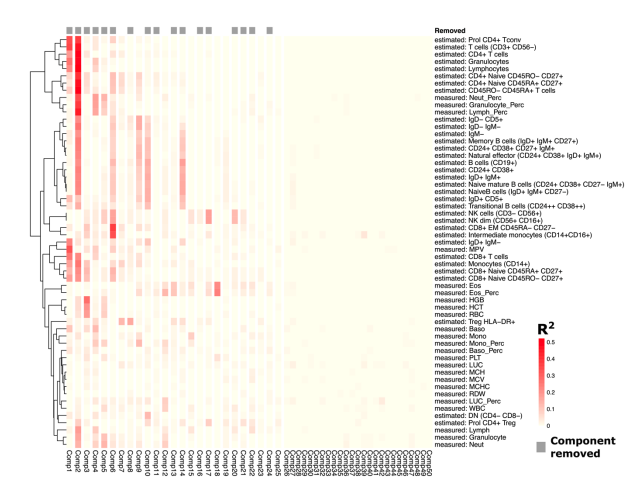
**

**Fig S1.** Correlation between the principal components (PCs) and imputed and measured cell counts. Correlation is calculated on the maximum number of available samples for each cell count metric (range N = 446-3,831). The grey blocks indicate the PCs that were regressed out from the expression data.

**Fig S2.** Comparison between the effects in the two sexes of the eQTLs in the 18 sex-specific eGenes. eQTLs showing a significant different effect in males and females are highlighted in red.

**Fig S3.** LD structure of *ZNF718* region. For each pair of SNPs, the r^2^ (red scale) and the D’ (clue scale) are reported.

**Fig S4.** Distribution of the expression values for the 18 sex-biased eGenes.

**Fig S5.** Cell type distribution in men and women. For the 33 imputed cell types we plotted their distribution in the two sexes. The indicators represent Wilcoxon p-values of below 0.05/33 (*), 0.01/33 (**) and 0.001/33 (***).

**Fig S6.** Distribution of the expression values after and before the PC correction. We plotted the distribution of the expression values of the 18 sex-specific eGenes after and before correcting for 25 principal components. The indicators represent Levene p-values of below 0.05/18 (*), 0.01/18 (**) and 0.001/18 (***).

**Fig S7.** QQ-plot for the 58 SNPs included in the BIOS Consortium dataset showing sex-biased effect in WHR or testosterone. In the y-axis, the Pvalue of the difference in effect in gene expression in the two sexes.


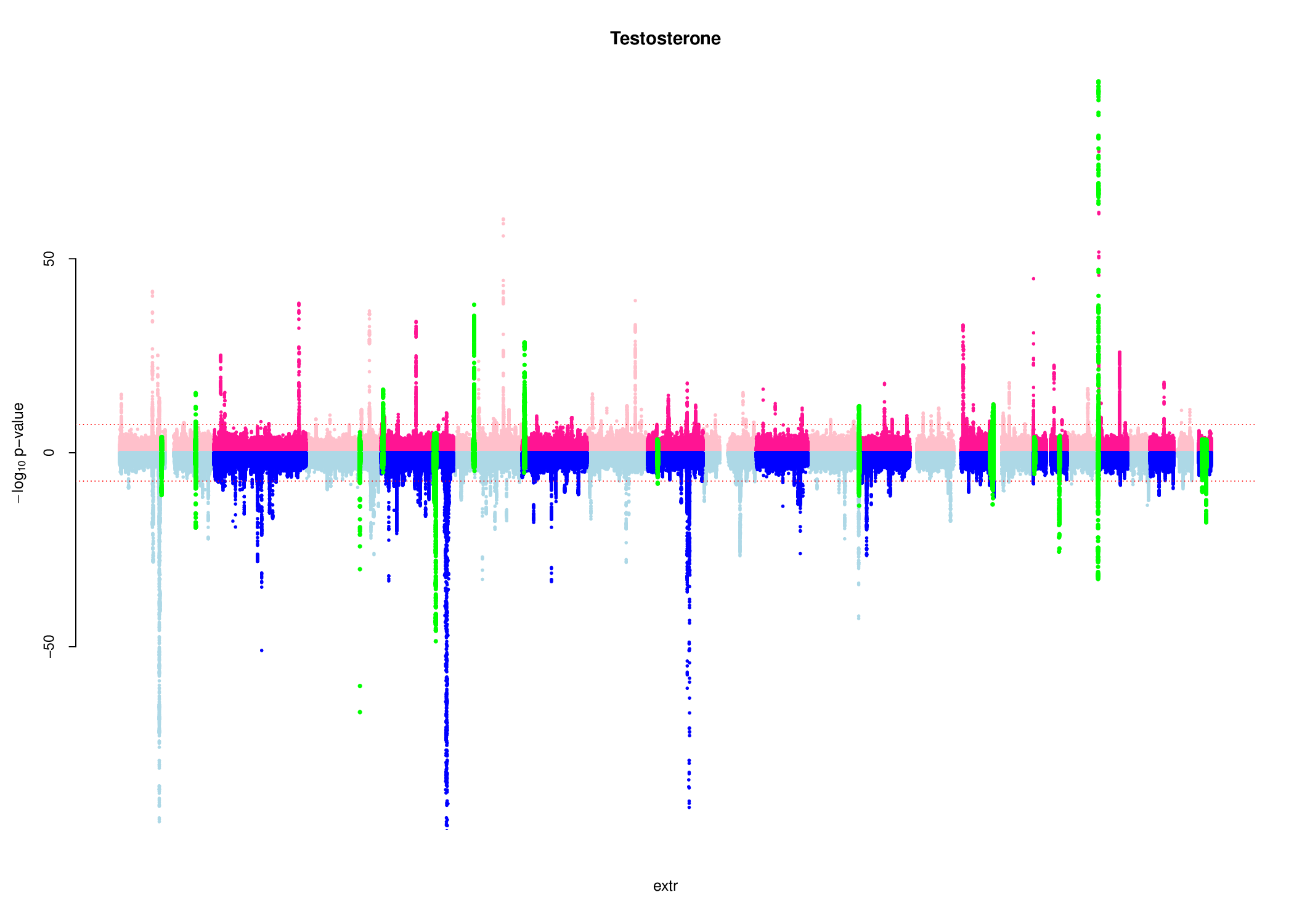


**Fig S8.** Miami plot for testosterone measured in UKBiobank (data from http://www.nealelab.is/uk-biobank/). SNPs are plotted on the x-axis according to their position on each autosomal chromosome against the P-values (shown as –/+ log_10_(P-value)) obtained upon testing for association in women (pink dots) and men (blue dots). Loci containing sex-specific causal genes are highlighted in green.


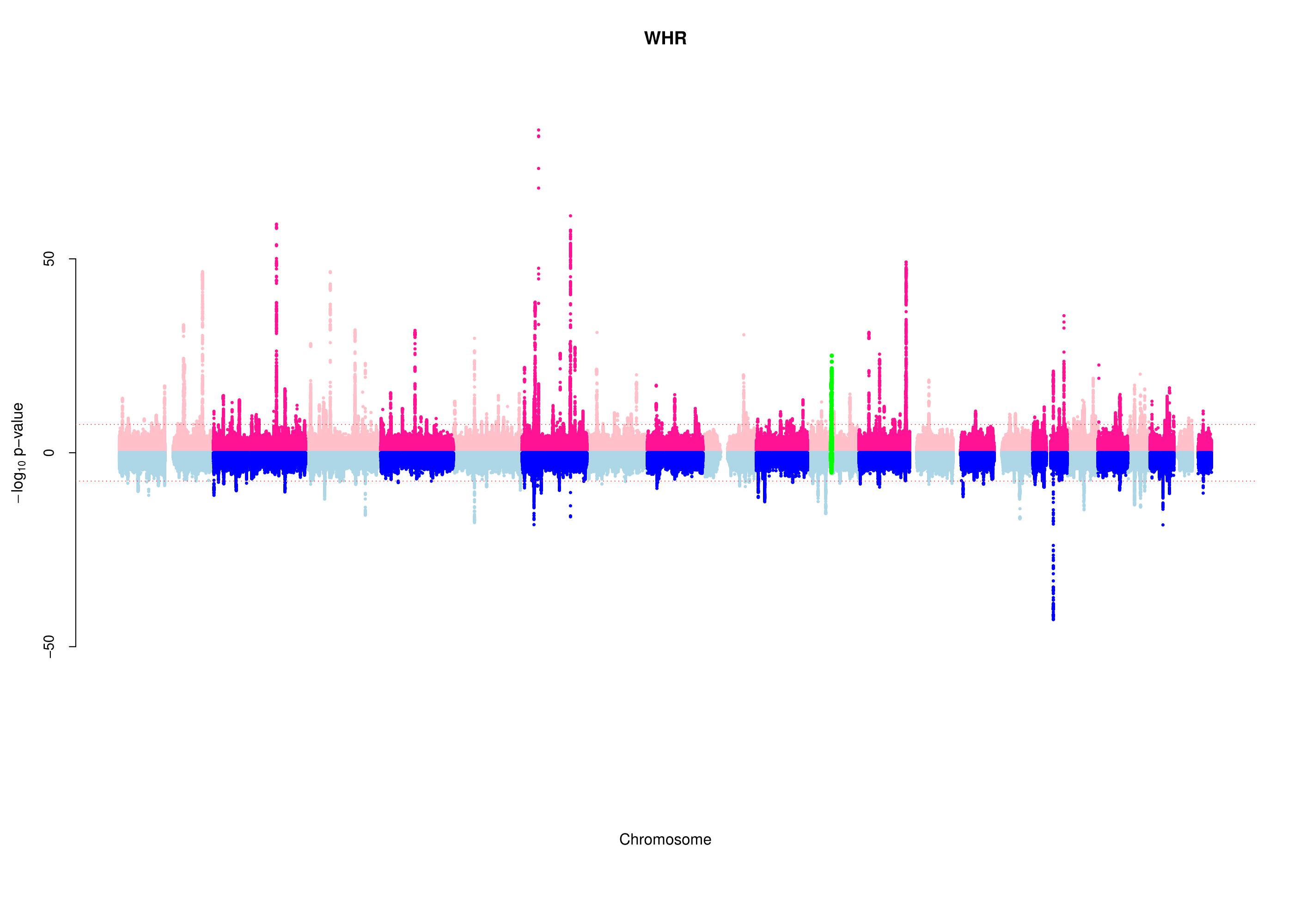


**Fig S9.** Miami plot for waist-to-hip ratio (WHR) measured in UKBiobank (data from http://www.nealelab.is/uk-biobank/). SNPs are plotted on the x-axis according to their position on each autosomal chromosome against the P-values (shown as –/+ log_10_(P-value)) obtained upon testing for association in women (pink dots) and men (blue dots). Loci containing sex-specific causal genes are highlighted in green.


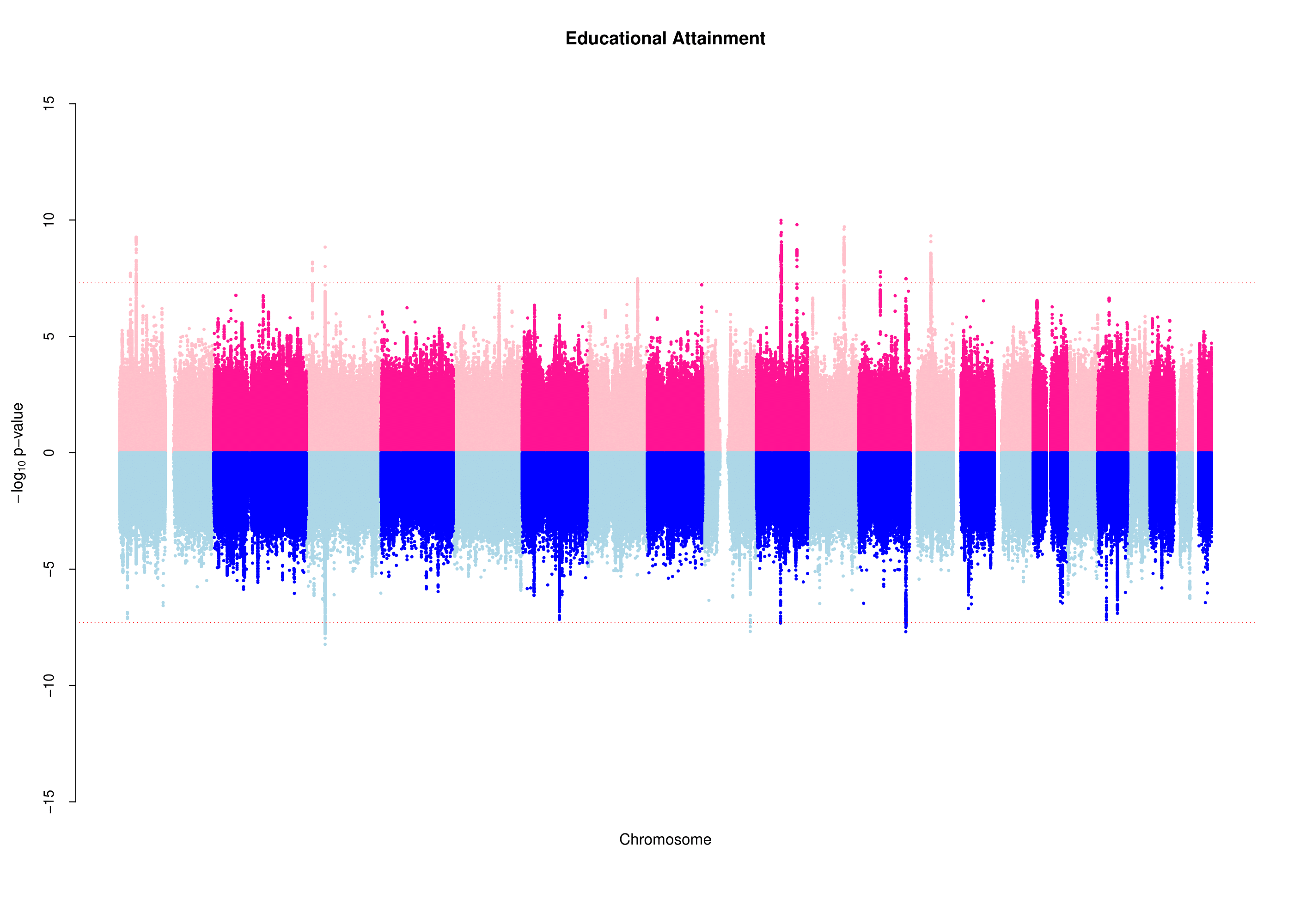


**Fig S10.** Miami plot for educational attainment measured in UKBiobank (data from http://www.nealelab.is/uk-biobank/). SNPs are plotted on the x-axis according to their position on each autosomal chromosome against the P-values (shown as –/+ log_10_(P-value)) obtained upon testing for association in women (pink dots) and men (blue dots).

**Fig S11.** Comparison of TWMR-causal effects estimated for WHR using sex-specific and combined eQTLs data.
